# PR3ANCA Related Cerebral Vasculitis in Ulcerative Colitis Presenting with Orbital Involvement: A Case Report with Review of Literature

**DOI:** 10.1155/2014/582094

**Published:** 2014-07-02

**Authors:** Athira Unnikrishnan, Shila Azodi, Nadeem Ansari, Megan Brown, Joshua Kamnetz, Robert C. Uchiyama

**Affiliations:** ^1^Department of Internal Medicine, University of Texas Southwestern Medical School Residency Programs at Austin, University Medical Center Brackenridge, Austin, TX 78701, USA; ^2^Department of Neurology, University of Texas Southwestern Medical School Residency Programs at Austin, University Medical Center Brackenridge, Austin, TX 78701, USA; ^3^Transitional Program, University of Texas Southwestern Medical School Residency Programs at Austin, University Medical Center Brackenridge, Austin, TX 78701, USA; ^4^School of Medicine, University of Texas Medical Branch, Galveston, TX 77555, USA

## Abstract

PR3 ANCA is a classic marker of granulomatosis with polyangiitis (GPA). There have been several recent reports of increased prevalence of PR3ANCA in ulcerative colitis (UC) patients, the clinical implication of which is not well defined. We are reporting a case of 27-year-old Caucasian male with 14-year history of UC presenting with unilateral proptosis, conjunctival congestion, and chemosis who developed acute hemiparesis within three days of hospital admission, followed by rapid neurological deterioration correlating with brain imaging findings. Serologically he had atypical PANCA with high PR3 antibody titer with a negative infectious workup. His cerebral angiogram was normal but the brain biopsy showed necrotizing vasculitis. He was diagnosed with PR3 ANCA mediated cerebral and orbital vasculitis associated with UC. Treatment was initiated with high dose steroids, plasmapheresis, and cyclophosphamide. He improved significantly with residual left hemiparesis.

## 1. Introduction

PR3 ANCA is a classic marker of granulomatosis with polyangiitis (GPA). There have been several recent reports of increased prevalence of PR3ANCA in ulcerative colitis patients, the clinical implication of which is not well defined [1]. In small cohort studies PR3 ANCA has been associated with extensive colitis and shorter disease duration without any gender predilection [2]. However, the incidence of small vessel vasculitis in PR3 ANCA positive UC patient cohort is not known. We are reporting the first case of PR3ANCA associated fulminant cerebral vasculitis in an ulcerative colitis patient.

## 2. Case Summary

A 27-year-old, right-handed Caucasian male with 13-year history of UC with primary sclerosing cholangitis presented to the ER with acute onset of left sided headache, left eye proptosis, erythema, and painful eye movements without any visual changes. He reported a recent trip to Spain and denied trauma, sick contacts, urinary symptoms, fever, weight loss, sexually transmitted diseases, or similar illness in family and friends.

He was diagnosed with UC with primary sclerosing cholangitis at age of 14 following chronic diarrhea, weight loss, guaiac positive stools, and elevated GGT. The colonoscopy then showed pancolitis extending throughout the colon with no evidence of granuloma. Liver biopsy showed onion skinning around the bile ducts. He was initially treated with steroids, mesalamine, and ursodiol. His last flare was a year ago. He had 4-5 blood streaked loose stools per day treated with tapering dose of steroids. Since then he has had no further UC flare and has been on mesalamine and ursodiol.

**Figure 1:**
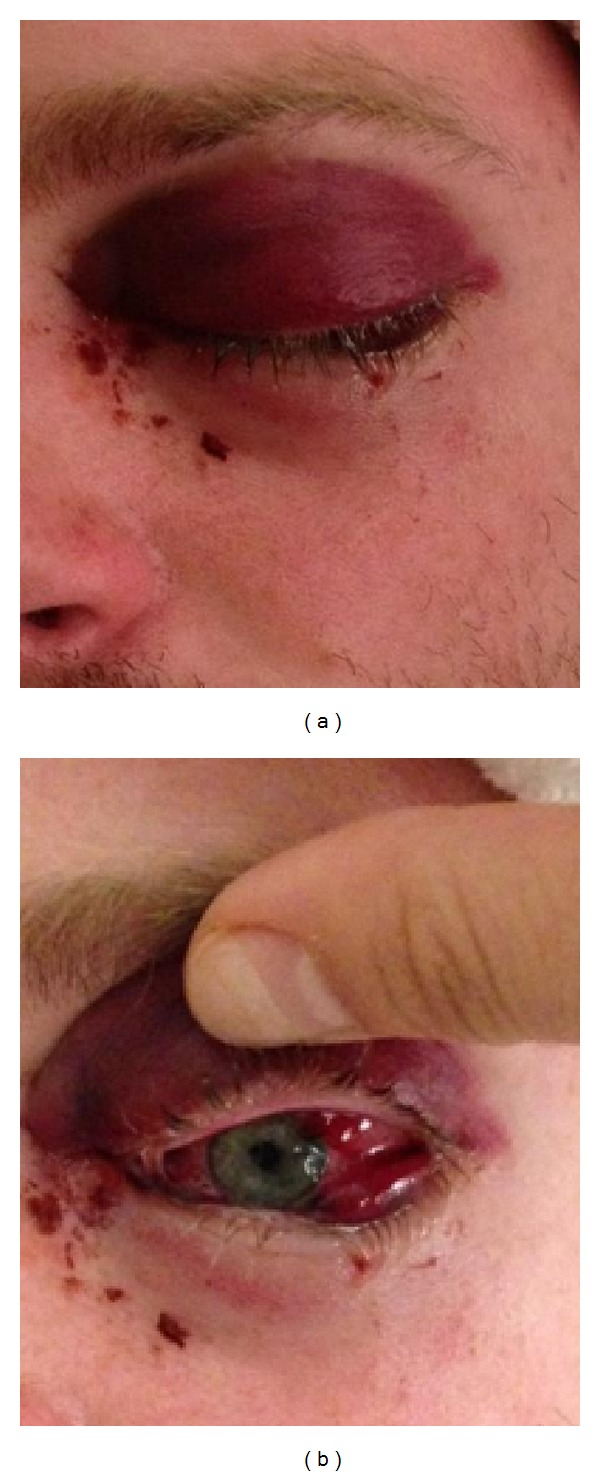
Day 1.

**Figure 2:**
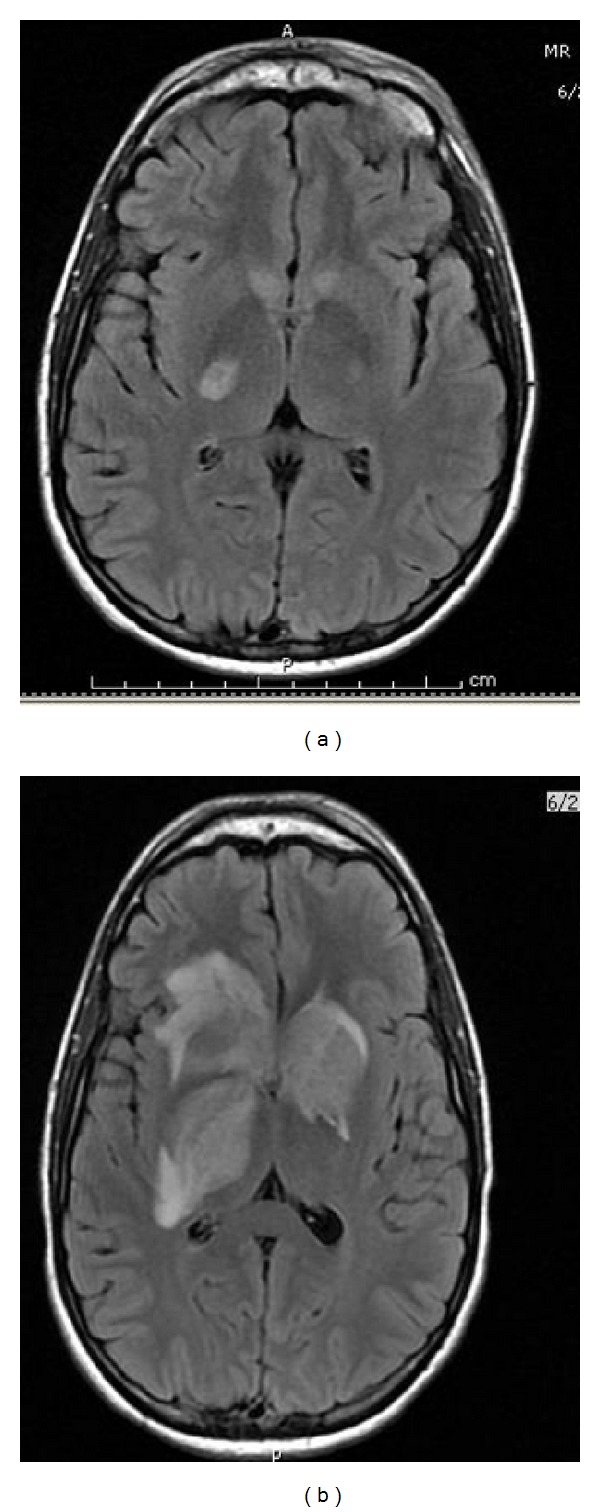
(a) Day 3. (b) Day 4.

Clinical examination revealed left eye ecchymosis, hemorrhagic chemosis, unilateral proptosis, with no evidence of uveitis, and normal fundus (see Figures 1(a) and 1(b)). Magnetic resonance imaging (MRI) of the face and orbit showed left eye proptosis with inflammation of the extraocular muscle and the periorbital tissue with normal brain and cavernous sinuses. He was treated with high dose steroids (one gram IV methyl prednisone for 3 days) and empiric antibiotics for orbital inflammatory syndrome and an autoimmune and infectious workup was started. His eye symptoms improved on steroids. However, on the third day of hospital admission, he complained of left arm weakness and numbness. A repeat MRI brain showed an interval development of multifocal deep gray nuclei signal abnormalities including diffusion restriction in the caudate nuclei, right globus pallidus, and posterior limb of the internal capsule (see Figure 2(a)). His CSF was essentially normal with 2 WBCS, normal protein, and glucose with few RBCs. He was empirically started on acyclovir. Within the next 36 hours, he rapidly worsened neurologically, became encephalopathic with left side hemiparesis, and had to be intubated for airway protection. His subsequent MRI brain showed further progression of lesions involving the caudate nuclei, thalami, striatum, and brainstem with new areas of hemorrhage and enhancement (Figure 2(b)). Cerebral arteriogram did not show any vasculitis.

Laboratory workup revealed mild leukocytosis with normal liver function tests, ESR, CRP, and thyroid function test. His laboratory findings were negative for HIV, hepatitis, toxoplasmosis,* Saccharomyces cerevisiae*,* Ehrlichia*,* Brucella*, Rocky Mountain spotted fever, Q fever, and Lyme disease. His CSF was negative for herpes simplex viruses 1 and 2, Adenovirus,* Enterovirus*,* Cryptococcus*, and* Mycobacterium tuberculosis*. His CSF and blood flow cytometry were negative for lymphoma/leukemia cells. Blood, stool, urine, and sputum cultures were sterile. An eye swab was negative for* Chlamydia trachomatis*. Skin biopsy was normal. Echocardiogram and CT scan of chest, abdomen, and pelvis were unremarkable. IgG4 was normal. Anticardiolipin antibody and beta 2 glycoprotein were both mildly elevated. He was treated empirically with antibiotics, antivirals, and steroids.

**Figure 3:**
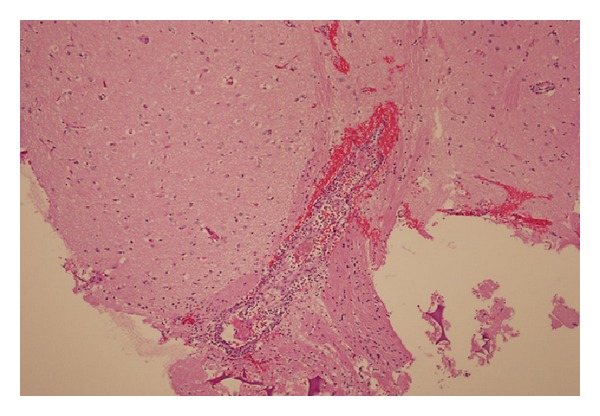
Brain biopsy.

He had an ANA titer of 1 : 40 and PANCA titer of 1 : 320; his PR3 antibody was 157 AU per milliliter (normal range, 0 to 19). The brain biopsy showed necrotizing vasculitis (Figure 3) with no evidence of granuloma. Absence of granuloma, in addition to normal lung, kidney, and sinuses ruled out GPA. Based on the serology and brain biopsy results we diagnosed PR3ANCA mediated cerebral and orbital vasculitis. He was treated similar to GPA involving the brain and eye with cyclophosphamide, plasmapheresis, and high dose of steroids. He dramatically improved with residual hemiparesis and was discharged to rehabilitation.

## 3. Discussion

The atypical PANCA seen in UC usually represents antibodies against lactoferrin, cathepsin, BPI, or elastase. PR3ANCA, antinuclear cytoplasmic antibody against proteinase3, the classic serological marker in granulomatosis with polyangiitis (GPA), has been noted to have an increased prevalence in ulcerative colitis. There has been a suggestion to use PR3ANCA as a serological marker for UC in the evaluation of IBD [1]. The reported incidence of PR3 ANCA in UC varies from 4 to 43% [1]. In small cohort studies PR3 ANCA has been associated with extensive colitis and shorter disease duration (average of 12 ± 2 years) without any gender predilection [2]. However, the incidence of small vessel vasculitis in PR3 ANCA positive UC patient cohort is not known. It is well established that in GPA PR3 antibodies with CANCA cause aggressive vasculitis which can rarely involve the eye and brain. To the best of our knowledge to date there have been no case reports of PR3ANCA mediated cerebral and orbital vasculitis in UC patients.

Cerebrovascular manifestations are seen in 0.12–4% of inflammatory bowel disease patients [17]. These include arterial and venous thrombosis, leukoencephalitis, seizures, and vasculitis. There have been only fifteen case reports of possible cerebral vasculitis in ulcerative colitis. Among these fifteen reported cases (Table 1) eleven patients had definite vasculitis based on histopathology, angiogram, or serology. Six patients had cerebral angiogram studies suggestive of vasculitis with multiple small and medium vessel narrowing, three had brain biopsy/autopsy confirming necrotizing vasculitis, one had MPO mediated systemic vasculitis with multiple brain infarcts, and one had biopsy proven skin vasculitis with multiple brain infarcts. The remaining four patients had evidence of multiple ischemic infarcts on brain imaging and vasculitis was suggested as a possible etiology based on clinical evidence. In these case series only one of the patients had MPO associated vasculitis. The limited autoimmune work up in the rest of the cases was negative. None of these patients had eye involvement. Most of these patients were treated with steroids alone or in combination with immunosuppressive therapy. Seven patients had complete recovery of neurological deficits, four patients had partial recovery, and one patient died and the diagnosis was made on the autopsy. Our patient had mild increase in antiphospholipid antibodies similar to the MPO associated vasculitis patient [6]. The elevation in these antibodies was likely a reflection of the vascular damage with vasculitis instead of the primary etiology.

The eye manifestations of UC commonly include uveitis, scleritis, and conjunctivitis which occur in about 1–6.3% of patients [18]. There are four case reports of orbital inflammatory syndrome reported in UC, of which three patients had orbital myositis and one had orbital pseudotumor [18, 19]. It is unclear if orbital vasculitis was the underlying etiology. Clinically our patient had severe orbital ecchymosis and conjunctival hemorrhagic chemosis which was atypical for an idiopathic orbital inflammatory syndrome. MRI showed extensive inflammatory changes in the left orbit including the periorbital soft tissue and the extraocular muscles. The brain biopsy with the positive vasculitis markers suggests the orbital inflammatory syndrome in our patient was secondary to orbital vasculitis.

Our experience in this patient suggests that PR3ANCA positivity can cause rapidly progressive cerebral vasculitis and orbital vasculitis in UC patients similar to GPA. Although there have been cases reported of ulcerative colitis later developing GPA [20] and some suggestion of an overlap between the entities there is no established relationship between GPA and UC [21]. Our patient clearly does not satisfy the diagnostic criteria to diagnose GPA.

## 4. Conclusion

Our case of cerebral vasculitis associated with ulcerative colitis is the fourth histopathologically proven case in the literature. There are no reports of orbital vasculitis and cerebral vasculitis coexisting in a patient with ulcerative colitis. This is the first case report of PR3ANCA mediated cerebral vasculitis in a UC patient presenting with orbital involvement. The fulminant course of the cerebral vasculitis in our patient highlights the need for increased awareness of the possibility of PR3ANCA associated vasculitis in UC patients to facilitate early diagnosis and treatment. We need further long term cohort studies of the subset of UC patients with PR3 ANCA to define the role of PR3 ANCA in UC and to establish any significant increase in vasculitis in this cohort. We could also study if there is any increase in GPA in this patient cohort.

## Figures and Tables

**Table 1:** Ulcerative colitis with cerebral vasculitis.

Author	Age	Clinical presentation	Cerebral biopsy	MRI and vascular studies	Serology	Treatment	Outcome
Nomoto et al. [3]	18/F	Diagnosed with UC at age 15, presented with headache, transient confusion	No	MRA: diffuse narrowing cerebral arteries with multiple segmental stenoses; common carotid and subclavian artery narrowing with irregularities	MPO-ANCA, PR3-ANCA within normal range	Prednisone	Resolution of neurological deficits

Pandian et al. [4]	35/F	Unknown duration UC, presented with right side weakness, unsteady gait	No	Restricted diffusion in left ACA territory; angiogram shows intracranial vessels with multiple areas stenosis and dilatation	Not reported	None	Not available

Nelson et al. [5]	18/M	One month after diagnosis UC presented with generalized tonic clonic seizures and became comatose	Yes; acute necrotizing vasculitis involving meningeal and cortical blood vessels with affected vessels showing fibrinoid necrosis with acute inflammatory cell infiltration	CT with multiple bilateral cerebral low density areas enhancing with contrast	ANA negative	Prednisone, cyclophosphamide	Resolution of neurological deficits

Panani et al. [6]	51/M	Eight-year diagnosis of UC presented with febrile illness, rash, acute deterioration	No; skin punch biopsy-lesions on the small vessels suggesting a possible systemic disease	CT head-ischemic lesions in white matter	p-ANCA MPO positive, anticardiolipin elevated	Prednisone, cyclophosphamide	Resolution of neurological deficits

Nemoto et al. [7]	69/F	Sensorineural deafness, ptosis, peripheral facial palsies, hyperreflexia all ext. and later diagnosed with UC	No	T2 hyperintensities in midbrain, pons, bilateral cerebral white matter; no vascular study	ANA negative	Corticosteroids	Improved but still had deafness and worsening cerebral deep white matter lesions without any new clinical signs

Druschky et al. [8]	37/M	Eight-year history of UC, weakness right arm, slurred speech, rapidly developing confusion	Not brain (upper arm skin biopsy showed perivascular infiltration with inflammation)	T2 hyperintensities periventricular and cerebellar, spinal cord; no vascular study	ANA, c-ANCA, p-ANCA negative	Corticosteroids, azathioprine, and cyclosporine A	Complete resolution

Dejaco et al. [9]	58/M	Diagnosed with UC at age of 29, hemiparesthesia of face and left and right side body intermittently	No	T2 hyperintensities of centrum semiovale (reported as typical of microangiopathy associated with vasculitis)	c-ANCA, p-ANCA negative	Prednisolone, ASA	Complete recovery

Masaki et al. [10]	19/F	Within 2 weeks presentation of bloody diarrhea developed generalized convulsive seizures and AMS; dysarthria, numbness of tongue and extremities	No	T2 and FLAIR multiple hyperintensities in the corticomedullary; enhancing lesions; cerebral angiogram: faint staining in parietooccipital area	c-ANCA, p-ANCA negative	Prednisolone, dextran, and colon resection	Complete recovery

Bonrath et al. [11]	72/M 61/F	Number 1 acute UC flare with AMS; number 2 active UC with acute left hemiparesis	One brain biopsy showed postischemic changes, inconclusive	Number 1 MRI brain showed multiple perivascular signal changes and infarcts; number 2 MRI also consistent with vasculitis	p-ANCA, c-ANCA, MPO, and Pr3 negative in both cases		

Carmona et al. [12]	47/M	Developed UC 7 years prior, presented with right motor hemiparesis and aphasia	Yes (autopsy), small and medium size vessel showed necrotizing angiitis	CT head showed low attenuation in left parietal and occipital regions	Not reported	Decadron, mannitol	Death

Glotzer et al. [13]	18/M	Diagnosed with UC 10 months prior, presented with left hemiparesis, hemianopia, AMS	Yes; necrotic mostly white matter with polymorphonuclear lymphocytes perivascularly	Carotid angiogram showing displacement of ACA, MCA with parietooccipital mass	Not reported	Erythromycin, clarithromycin, and amphotericin B	Complete neurological recovery

Edwards [14]	28/M	2 months after diagnosis UC presented with right arm weakness, right facial paresis, GTCS	No	Bilateral carotid angiogram showing mulivessel segmental narrowing in small and medium arteries	ANA negative	Dexamethasone	Residual left hemiparesis

Friol-Verceletto et al. [15]	45/F	14-year diagnosis of UC with spastic hemiparesis	No	Abnormal angiography	ESR elevated	Not described	Not described

Karacostas et al. [16]	32/F	At time of diagnosis UC developed left hemiparesis, AMS, generalized seizures	No	CT head number 1 right frontal pole hypodensity; number 2 showed hemorrhagic transformation of the ischemic infarct and white matter edema; right side carotid angiogram reported as patent vessels	ANA and lupus anticoagulant negative	Prednisone	Significant neurological
